# Health‐Related Quality of Life One Year After Intensive Care Unit Admission for COVID‐19: A Retrospective, Cross‐Sectional, Longitudinal Observational Study

**DOI:** 10.1002/hsr2.70507

**Published:** 2025-02-24

**Authors:** Carina M. Samuelsson, Netha Hussain, Avril Drummond, Carina U. Persson

**Affiliations:** ^1^ Department of Occupational Therapy and Physiotherapy Sahlgrenska University Hospital/Östra Gothenburg Sweden; ^2^ Department of Radiology Sahlgrenska University Hospital Gothenburg Sweden; ^3^ Faculty of Medicine and Health Sciences, University of Nottingham Nottingham UK; ^4^ Department of Clinical Neuroscience, Rehabilitation Medicine Institute of Neuroscience and Physiology, Sahlgrenska Academy, University of Gothenburg Gothenburg Sweden; ^5^ Department of Medicine, Geriatrics, and Emergency Medicine Centre for Lifestyle Intervention, Sahlgrenska University Hospital/Östra Gothenburg Sweden

**Keywords:** COVID‐19, health‐related quality of life, health status, intensive care unit

## Abstract

**Background and Aims:**

Although sequelae are commonly reported in intensive care unit (ICU) survivors following COVID‐19, country‐specific long‐term health‐related quality of life (HRQoL) studies are scarce. Therefore, the aims were to describe HRQoL and to identify early factors associated with impaired HRQoL 1 year after ICU admission following COVID‐19.

**Methods:**

This retrospective, cross‐sectional, longitudinal observational study assessed HRQoL 1 year after ICU admission for COVID‐19 during the first wave of the pandemic. HRQoL was measured using the EuroQol 5 Dimensions 3 Levels (EQ‐5D‐3L) questionnaire, which covers Mobility, Self‐Care, Usual Activities, Pain/Discomfort and Anxiety/Depression, along with the EuroQol visual analogue scale (EQ‐VAS). Univariable and multivariable logistic regression analyses were performed to identify associations between HRQoL and the independent variables.

**Results:**

A total of 105 participants (median age 58 [interquartile range {IQR}: 51–66] years) completed the EQ‐5D‐3L. Over two‐thirds (*n* = 73, 69%) reported moderate or extreme problems related to Pain/Discomfort and half (*n* = 53, 51%) reported problems related to Anxiety/Depression. The mean EQ‐5D‐3L value index was 0.83 (standard deviation ±0.13). For the EQ‐VAS (*n* = 103), the median score was 70 (IQR: 59–80). Diabetes mellitus was associated with impaired HRQoL in Self‐Care (odds ratio [OR]: 7.51, 95% confidence interval [CI]: 1.77–31.92) and longer length of stay in ICU was associated with impaired HRQoL in both Usual Activities (OR: 1.03, 95% CI: 1.01–1.05) and Pain/Discomfort (OR: 1.08, 95% CI: 1.03–1.13). Also, younger age (OR: 0.96, 95% CI: 0.92–1.00) and female sex (OR: 0.21, 95% CI: 0.06–0.70) were associated with impaired HRQoL in Pain/Discomfort.

**Conclusion:**

The prevalence of impaired HRQoL 1 year after ICU care due to COVID‐19 is a public health concern. Our findings imply that COVID‐19 ICU survivors, and particularly those with diabetes, should be followed up beyond 1 year to identify those in need of continued mental and physical health care and rehabilitation.

## 1 Background

From March 2020 until May 2023, coronavirus 2019 (COVID‐19) was classified as a pandemic [1]. A large proportion of COVID‐survivors have been affected by long‐term sequelae, referred to as post‐COVID‐19 [2]. In intensive care unit (ICU) survivors with COVID‐19, fatigue [3, 4, 5], cognitive impairment [4, 5], dyspnoea [6, 7] and restricted exercise capacity [8, 9] have all been reported in the year after admission. However, it is not clear what impact these symptoms have specifically on individuals' health‐related quality of life (HRQoL) [10, 11]. There is also limited country‐specific data regarding the long‐term prevalence of impaired HRQoL at 1 year following ICU admission for COVID‐19 [4, 12]. It is important to bridge this knowledge gap since there are already population variabilities between countries, as for example in population density, median age, land area and gross domestic product [13].

Prior studies on HRQoL have largely focused on individuals with varying severity of COVID‐19 and with varying follow‐up times of up to 1 year [10, 14, 15, 16, 17, 18, 19, 20]. There is sparse data exclusively on 1‐year follow‐up of patients with severe COVID‐19 admitted to an ICU [21]. Several previous studies of COVID‐19 survivors have reported that approximately 50%–60% had impaired HRQoL 1 year after ICU care [4, 5, 22, 23, 24].

To enable identification of at‐risk individuals of impaired HRQoL already during the ICU care, it is important to identify the early predictors. However, research on risk factors for impaired HRQoL at 1 year after ICU care following severe COVID‐19 is scarce. Previous studies have shown that both male [22] and female sex [12, 23], and comorbidities [12] have been identified as predictors. To the best of our knowledge, the predictors for specific factors affecting the dimensions of HRQoL related to the survey instrument EuroQol 5 Dimensions (EQ‐5D) [25], i.e., Mobility, Self‐Care, Usual Activities, Pain/Discomfort, and Anxiety/Depression, have not previously been investigated. This knowledge will be of value for primary healthcare practitioners and policy makers to facilitate early rehabilitation in addition to long‐term follow‐up and support for individuals at‐risk of impaired HRQoL.

Along the pandemic, the disease has changed [26]. However, in the post‐COVID era where physicians face the long‐term consequences of the disease, it is clinically relevant and important to address these gaps related to the unavailability of country‐specific prevalence data and the limited number of prediction studies. The aims of this study therefore were to describe the HRQoL and to identify early factors associated with impaired HRQoL 1 year after ICU admission following severe COVID‐19. We hypothesized that impaired HRQoL was common 1 year after admission to ICU due to COVID‐19. Based on previous research, we hypothesized that older age [19], male sex [22], longer length of stay in ICU [19] and comorbidities [27] were associated with impaired HRQoL.

## 2 Methods

This study, the Gothenburg Recovery and Rehabilitation after COVID‐19 and Intensive Care Unit (GOT‐RECOV‐19 ICU), had a retrospective, cross‐sectional, and longitudinal observational design. The GOT‐RECOV‐19 ICU study was registered at “FoU i Sverige” (Research and development in Sweden, researchweb.org) on May 28, 2020 (ID number: 274477) and has three previous related publications [3, 28, 29]. Ethical approval was obtained on September 24, 2020 (ID number 2020‐03264) from the Swedish Ethical Review Authority. An additional approval was obtained on June 29, 2021 (ID number 2021‐03220) for the sending of the remainder emails to participants. The study was conducted in accordance with the Strengthening the Reporting of Observational Studies in Epidemiology (STROBE) guidelines [30].

The inclusion criteria were individuals, 18 years of age or older, diagnosed with COVID‐19 (diagnostic code U07.1, according to the classification system ICD‐10) and who were still alive 1 year after admission to one of the five ICUs at Sahlgrenska University Hospital between March 1st to June 30th, 2020. The exclusion criteria were individuals not living in Gothenburg or its surrounding municipalities or not registered as a resident of Sweden in the Swedish Population Register. Individuals who met the inclusion criteria were sent a postal request to participate, including clinical assessments and questionnaires. At most, two postal reminders were sent to those who did not respond. In the second reminder, potential participants were given an option to respond only to the questionnaires. All participants gave written informed consent before their inclusion.

The clinical characteristics of the participants at baseline were obtained from medical records. The independent variables (i.e., potential predictors) were personal factors (i.e., age and sex), ICU diagnosis (i.e., acute respiratory distress syndrome (ARDS) and sepsis), comorbidities (i.e., diabetes mellitus and heart disease [chronic heart failure and coronary heart disease]) and clinical parameters (i.e., mechanical ventilation [intubation] and length of stay in ICU).

Starting in March 2021, data on the primary outcome, HRQoL, was collected consecutively 1 year after ICU admission due to COVID‐19. HRQoL was assessed using the EQ‐5D‐3L questionnaire, a standardized tool developed by the EuroQol Group to measure HRQoL [31]. The questionnaire consists of two sections: the EQ‐5D‐3L descriptive system [25] and the EQ‐5D‐3L visual analogue scale (EQ‐VAS) [25]. The dependent variables were the five dimensions describing HRQoL in the EQ‐5D‐3L (i.e., Mobility, Self‐Care, Usual Activities, Pain/Discomfort, Anxiety/Depression). Each of the five dimensions has a statement with three response categories: no problems, moderate problems, and extreme problems, labeled level one to three, respectively. For each dimension, the participants chose the most appropriate statement regarding their present health status [32]. A fully completed EQ‐5D‐3L, consisting of the five options, results in a unique health state represented by five digits. The health state 11111 indicates no problems in any of the five dimensions, while the health state 33333 indicates extreme problems in all the dimensions [32]. The health state of each participant was then converted into the EQ‐5D‐3L value index, a score between 0 and 1 [33]. The EQ‐5D‐3L value index was obtained by experience‐based value set, using reference data from 45,477 individuals of the Swedish general population [33]. The participants were also asked to score their self‐perceived present health status using the EQ‐VAS. This is a vertical scale ranging from “The worst health you can imagine” (0) to “The best health you can imagine” (100) and is a valid and reliable questionnaire for describing and valuing health [25]. It has been employed in prior COVID‐19 studies [19, 22, 23, 34]. The EQ‐VAS has five severity levels from very poor to very good health, based on cohort data from the general Swedish population [33].

### 2.1 Statistics

Descriptive data on participant characteristics and data derived from the EQ‐5D‐3L and the EQ‐VAS were presented as means and standard deviations (SDs) or as medians and interquartile ranges (IQRs) for both interval and ordinal data. Nominal data was presented as frequencies (n) and percentages (%). The statistical analyses were performed using the IBM Statistical Package for Social Sciences (SPSS) software, version 28.

Mann–Whitney *U*‐tests were performed to compare age, sex, and length of stay at ICU between two groups: the 105 individuals who participated in the study and those who declined participation or did not respond to the study request. Univariable logistic regression analyses were performed to identify any association between the five dependent variables, Mobility, Self‐Care, Usual Activities, Pain/Discomfort and Anxiety/Depression, in the EQ‐5D‐3L (one analysis for each dimension was conducted) and the independent variables. In the regression analyses, based on the answers in every dimension, the EQ‐5D‐3L results were dichotomized [32] to “no problems” (Level 1) versus “any problems” [i.e., “moderate problems” (Level 2) or “extreme problems” (Level 3)] [25]. To be included in the multivariable logistic regression analysis, a cut‐off value of *p* < 0.10 was applied in the univariable analyses, however, age and sex were included as confounders regardless of their *p*‐values.

Any multicollinearity between the independent variables was determined using Spearman's rank correlation, where a correlation coefficient ≥ 0.7 were considered multicollinear [35]. Model fit for the multivariable logistic regression was assessed with the Hosmer–Lemeshow test, and improvements in model fit were evaluated using Cox & Snell and Nagelkerke pseudo *R* ^2^ values. The results of the univariable and multivariable logistic regression analyses were presented as odds ratios (ORs) with 95% confidence intervals (CIs). A *p*‐value ≤ 0.05 (two‐tailed) was considered statistically significant in the final multivariable models.

## 3 Results

**Figure 1:**
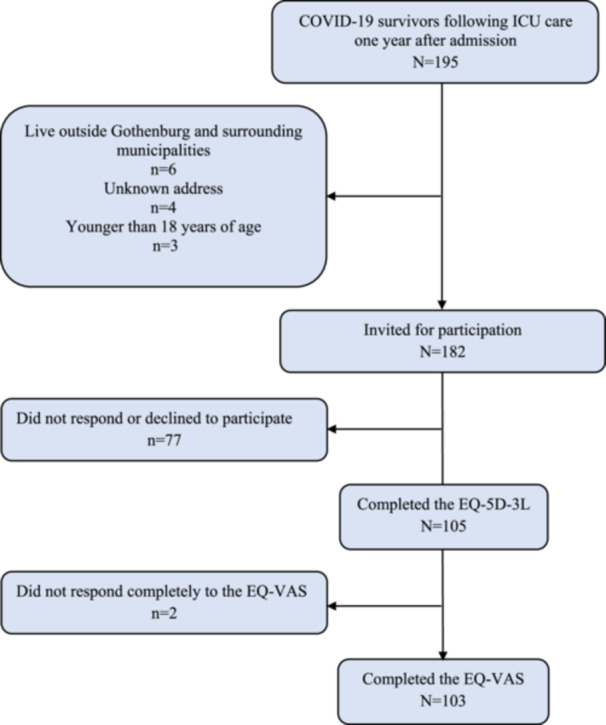
Enrollment in the study.

Of the 259 patients who were admitted to the relevant ICUs during the study period, 64 died within the year after admission. Of the 182 invited ICU survivors who met the inclusion criteria, 105 (58%) responded to the EQ‐5D questionnaire and were included in the study. Figure 1 shows the inclusion process.

Table 1 presents the clinical characteristics of the 103 participants (two did not score their self‐perceived health status on the EQ‐VAS) 1 year after admission to the ICU, divided into four groups based on their EQ‐VAS score and severity levels of health status (i.e., worse than poor health, poor health, fair health, and good/very good health). The levels of health status are based on classification performed in previous research [33]. More than four out of five participants required mechanical ventilation, i.e., intubation, and three individuals were treated with extracorporeal membrane oxygenation (ECMO) therapy. The median age based on 105 participants at baseline was 58 (IQR: 51–66) years, nearly three‐quarters were younger than 65 years and 24% (*n* = 25) were women. The demographic characteristics at baseline for all the participants (*n* = 105) have previously been presented [3]. The non‐participants had a median age of 57 (IQR: 46–65) years, and 27% were women (not shown in table). In those who had an EQ‐VAS score between 20 and 41 (i.e., worse than poor severity health status), more than four out of five were younger than 65 years. In addition, they had a higher BMI, and all were affected with ARDS during their hospital stay and had a longer ICU stay compared with the participants who scored over 41. We found no significant difference between the 105 participants recruited into the study and the 77 individuals who did not respond or declined to participate, regarding age, sex, or length of stay at ICU.

**Table 1:** Clinical characteristics at baseline for the 103^a^ participants divided into four groups based on their EQ‐VAS score and severity levels of health status, from left to right: worse than poor health, poor health, fair health and good/very good health.

	EQ‐VAS score 20–41	EQ‐VAS score 42–65	EQ‐VAS score 66–83	EQ‐VAS score 84–100
Clinical characteristics at baseline	*n* = 13	*n* = 30	*n* = 38	*n* = 22
Age (years)	58 (50–62)	58 (52–64)	58 (49–68)	61 (51–71)
Age under 65 years	11 (84.6)	24 (80.0)	24 (63.2)	13 (59.1)
Sex, male	11 (84.6)	19 (63.3)	30 (78.9)	20 (90.9)
BMI, kg/m^2^ (*n* = 54)	33.5 (27.2–37.3)	26.9 (23.4–31.0)	31.3 (28.3–34.8)	28.4 (22.2–33.6)
ICU length of stay	24 (12–32)	16 (7–24)	15 (8–29)	11 (10–21)
Hospital length of stay (*n* = 102)	43 (25–63)	23 (16–49)	24 (16–46)	24 (15–34)
Employed full‐time at ICU‐admission	9 (69.2)	23 (76.7)	31 (81.6)	11 (50.0)
ARDS	13 (100.0)	14 (46.7)	18 (47.4)	11 (50.0)
Sepsis	7 (53.8)	12 (40.0)	23 (60.5)	13 (59.1)
Type of respiratory support				
Mechanical ventilation (i.e., intubated)	13 (100.0)	23 (76.7)	30 (78.9)	19 (86.4)
High flow oxygen therapy	0 (0.0)	5 (16.7)	7 (18.4)	3 (13.6)
Mask or nasal cannula	0 (0.0)	2 (6.7)	1 (2.6)	0 (0.0)
Comorbidities				
Asthma	2 (15.4)	4 (13.3)	1 (2.6)	1 (4.5)
Chronic heart failure	0 (0.0)	2 (6.7)	1 (2.6)	1 (4.5)
Chronic kidney disease	0 (0.0)	3 (10.0)	2 (5.3)	1 (4.5)
Chronic obstructive pulmonary disease	1 (7.7)	0 (0.0)	0 (0.0)	1 (4.5)
Coronary heart disease	3 (23.1)	8 (26.7)	13 (34.2)	3 (13.6)
Diabetes mellitus	3 (23.1)	10 (33.3)	8 (21.1)	2 (9.1)
Hypertension	4 (30.8)	16 (53.3)	17 (44.7)	6 (27.3)

*Note:* Data are presented as medians (IQR), frequencies (n), and percentages (%). The four severity levels of health status related to the EQ‐VAS score are based on previous research [33].

Abbreviations: ARDS, acute respiratory distress; BMI, body mass index; EQ‐VAS, EQ visual analogue scale; ICU, intensive care unit; IQR, interquartile range; SD, standard deviation.

a Two participants did not score their self‐perceived present health status on the EQ‐VAS.

**Figure 2:**
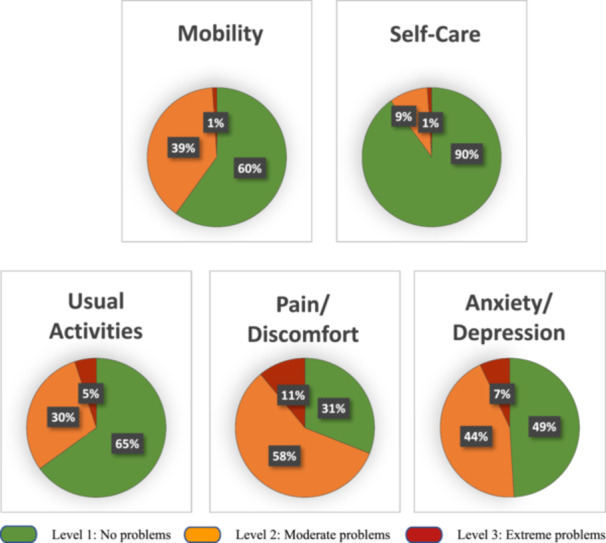
Self‐perceived health status of the 105 participants using the EuroQol 5 Dimensions 3 Levels (EQ‐5D‐3L) 1 year following intensive care unit admission. The data is presented as the proportions (%) of the participants reporting no problems, moderate problems, extreme problems across the five dimensions.

A summary of the EQ‐5D‐3L data with the proportions (%) of the three levels (no problems, moderate problems, and extreme problems) for each of the five dimensions for the participants is presented in Figure 2. The most common health state, reported by 21 participants (20%), was 11111, which refers to no problems in any health dimension. The second most common health state, reported by 15 (14%), was 11121, i.e., moderate problems related to Pain/Discomfort. None reported the health state 33333, i.e., none reported extreme problems in all the five dimensions. The most frequently reported problems (i.e., moderate, and extreme problems) were reported in Pain/Discomfort by more than two‐thirds (*n* = 73, 69%). More than half (*n* = 53, 51%) reported problems in Anxiety/Depression. One out of 10 participants reported problems related to Self‐Care and more than one‐third (*n* = 37, 35% and *n* = 42, 40%, respectively) reported problems related to Usual Activities and Mobility. Extreme problems were most prevalent in Pain/Discomfort and Anxiety/Depression, which were reported by 12 (11%) and 7 (7%) participants, respectively. Sixty‐four (61%) reported moderate or extreme problems in more than one dimension.

**Figure 3:**
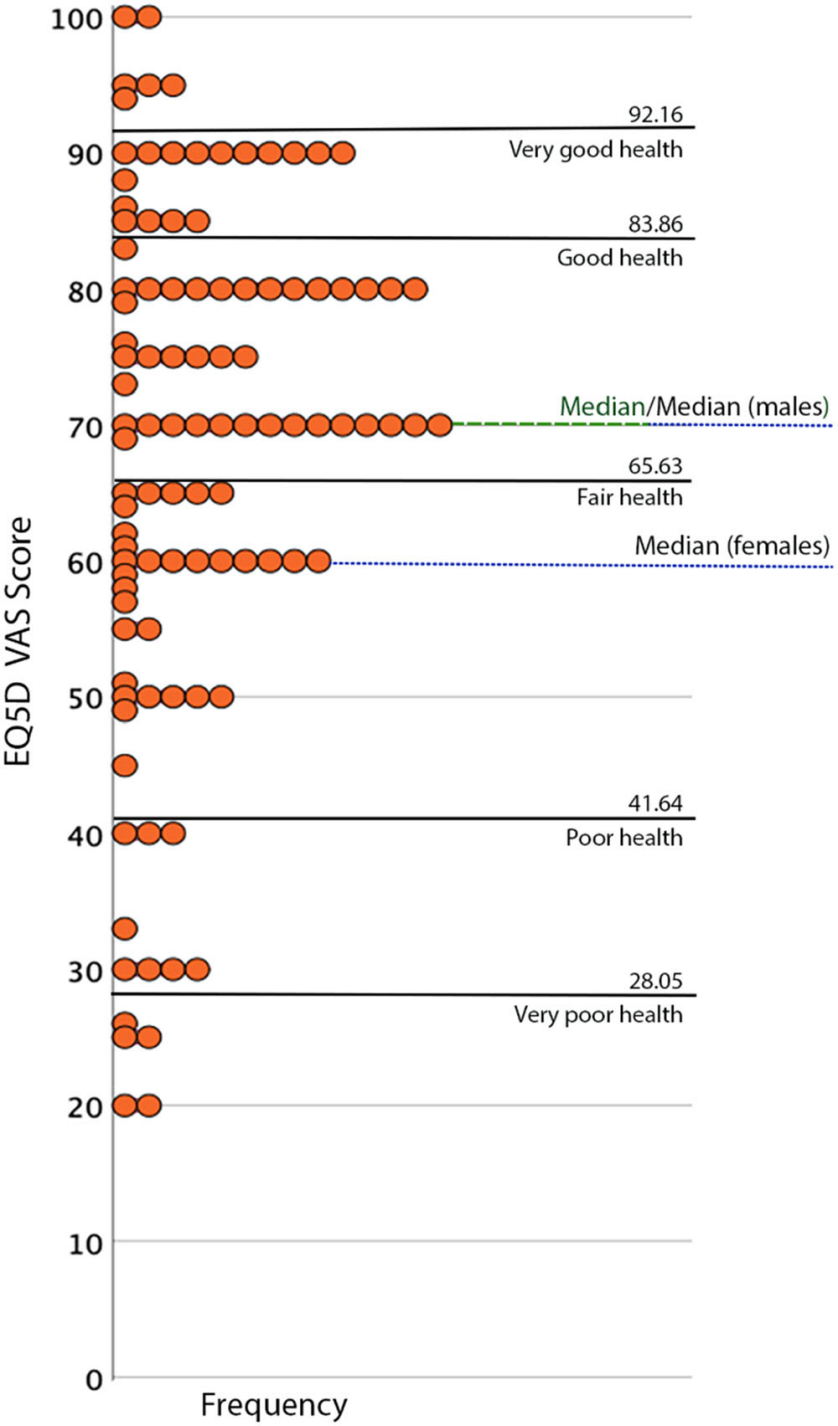
Self‐perceived present health status of the 103 COVID‐19 survivors with a completed EuroQol visual analogue scale 1 year following intensive care unit admission.

The mean and the median of the EQ‐5D‐3L value index was 0.83 (SD 0.13) and 0.87 (IQR: 0.71–0.93), respectively. For women and men, the mean value index was 0.84 (0.14) and 0.82 (0.13), respectively (not shown in table). Figure 3 shows a compilation of the scores for the self‐perceived present health status according to the EQ‐VAS for the 103 participants who completed the EQ‐VAS scale. The median EQ‐VAS score was 70 (IQR: 59–80) out of 100, which lies just above the level for a fair health status. For women and men, the median EQ‐VAS score was 60 (IQR: 50–80) and 70 (IQR: 60–84), respectively. The mean EQ‐VAS score was 67.6 (SD 19.2). Just over 1 in 10 (12%) participants had a health status worse than poor, while approximately one out of five (21%) had a health status of good or very good. Table 2 shows the results from the univariable logistic regression analyses for each of the five dependent variables. Diabetes mellitus was identified as a significant predictor of increased problems related to Self‐Care, and longer length of stay in ICU as a predictor of problems related to Usual Activities and Pain/Discomfort. Between the other two HRQoL‐dimensions, Mobility and Anxiety/Depression, and the independent variables, no statistically significant association were found. The multivariable regression analysis showed that diabetes mellitus [OR: 7.51 (95% CI: 1.77–31.92)], serves as a statistically significant predictor of impaired HRQoL in Self‐Care. Furthermore, in examining length of stay in the ICU as an independent variable, with Usual Activities and Pain/Discomfort as dependent variables, length of stay in ICU [OR: 1.03 (95% CI: 1.01–1.05), and 1.08 (95% CI: 1.03–1.13), respectively], was a statistically significant predictor for both outcomes. Additionally, younger age [OR: 0.96 (95% CI: 0.92–1.00)], and female sex [OR: 0.21 (95% CI: 0.06–0.70)], emerged as statistically significant predictors of impaired HRQoL in Pain/Discomfort in the multivariable regression analysis (shown in Table 3).

**Table 2:** Univariable logistic regression analyses, one for each of the five dependent variables, for early prediction of impaired HRQoL 1 year following admission to ICU due to COVID‐19 using the EQ‐5D‐3L for the 105 participants.

	Mobility		Self‐care		Usual activities		Pain/discomfort		Anxiety/depression	
Independent variables	Odds ratio (95% CI)	*p*	Odds ratio (95% CI)	*p*	Odds ratio (95% CI)	*p*	Odds ratio (95% CI)	*p*	Odds ratio (95% CI)	*p*
Age^a^	1.01 (0.98–1.05)	0.56	1.00 (0.94–1.05)	0.90	0.98 (0.95–1.02)	0.27	0.97 (0.94–1.01)	0.16	0.97 (0.94–1.00)	0.07
Male sex (Ref = female sex)	0.61 (0.26–1.61)	0.35	0.43 (0.11–1.65)	0.22	0.76 (0.30–1.93)	0.57	0.49 (0.17–1.45)	0.20	0.48 (0.19–1.22)	0.13
ARDS (Ref = No)	0.80 (0.37–1.75)	0.58	0.58 (0.15–2.17)	0.41	1.24 (0.55–2.77)	0.60	1.74 (0.75–4.03)	0.20	1.30 (0.61–2.81)	0.50
Sepsis (Ref = No)	0.73 (0.33–1.59)	0.43	0.58 (0.15–2.17)	0.41	1.56 (0.69–3.50)	0.29	1.15 (0.50–2.64)	0.75	1.04 (0.48–2.23)	0.93
Diabetes mellitus (Ref = No)	1.89 (0.77–4.81)	0.18	6.88 (1.75–27.07)	**0.006**	1.24 (0.48–3.20)	0.66	1.77 (0.59–5.27)	0.31	0.87 (0.35–2.20)	0.77
Heart disease (Ref = No)	1.04 (0.43–2.54)	0.93	0.30 (0.04–2.44)	0.26	0.89 (0.35–2.25)	0.81	0.54 (0.21–1.34)	0.18	0.47 (0.30–1.74)	0.47
Mechanical ventilation (Ref = No)	0.78 (0.29–2.07)	0.78	1.35 (0.36–5.09)	0.66	1.81 (0.60–5.46)	0.29	2.21 (0.81–6.01)	0.12	1.31 (0.49–3.49)	0.59
Length of stay in ICU^a^	1.02 (0.99–1.04)	0.07	0.98 (0.94–1.03)	0.48	1.02 (1.00–1.04)	**0.04**	1.05 (1.01–1.09)	**0.02**	1.00 (0.09–1.02)	0.92

*Note:* The five dependent variables (Mobility, Self‐Care, Usual Activities, Pain/Discomfort, and Anxiety/Depression) were dichotomized according to “no problems” versus “any problems” (i.e., moderate, or extreme problems). Significant values are given in **bold**.

Abbreviations: ARDS, acute respiratory distress syndrome; CI, confidence interval; EQ‐5D‐3L, EuroQol 5 Dimensions 3 Levels; HRQoL, health‐related quality of life; ICU, intensive care unit; *p*, *p*‐value.

a Risk by one unit increase.

**Table 3:** Multivariable regression analysis predicting impaired HRQoL in self‐care, usual activities, and pain/discomfort 1‐year post‐ICU admission for COVID‐19, adjusted for age and sex (*n* = 105).

Dependent variable—Self‐care		
Independent variables	Odds ratio (95% CI)	*p*
Age^a^	0.98 (0.92–1.04)	0.46
Sex (Ref = female sex)	0.46 (0.11–1.96)	0.30
Diabetes mellitus	7.51 (1.77–31.92)	**0.006**

Dependent variable—Usual activities		
Independent variables	Odds ratio (95% CI)	*p*
Age^a^	0.97 (0.94–1.01)	0.14
Sex (Ref = female sex)	0.49 (0.18–1.33)	0.16
Length of stay in ICU^a^	1.03 (1.01–1.05)	**0.02**

Dependent variable—Pain/discomfort		
Independent variables	Odds ratio (95% CI)	*p*
Age^a^	0.96 (0.92–1.00)	**0.05**
Sex (Ref = female sex)	0.21 (0.06–0.70)	**0.01**
Length of stay in ICU^a^	1.08 (1.03–1.13)	**0.002**

*Note:* For the models with the dependent variables self‐care, usual activities, and pain/discomfort, respectively: The Hosmer–Lemeshow goodness‐of‐fit test yielded significance levels of 0.483, 0.899, and 0.642. The Cox & Snell *R* ^2^ values were 0.084, 0.079, and 0.170, and the Nagelkerke *R* ^2^ values were 0.180, 0.109, and 0.240, respectively. The dependent variables (self‐care, usual activities and pain/discomfort) were dichotomized according to “no problems” versus “any problems” (i.e., moderate, or extreme problems). Significant values are given in **bold**.

Abbreviations: CI, confidence interval; EQ‐5D‐3L, EuroQol 5 Dimensions 3 Levels; HRQoL, health‐related quality of life; ICU, intensive care unit; *p*, *p*‐value.

^a^ Risk by one unit increase.

## 4 Discussion

We aimed to describe HRQoL, and to identify any factors that were associated with impaired HRQoL, 1 year after ICU admission following severe COVID‐19. Using the EQ‐5D‐3L, we found that more than two‐thirds of the participants reported moderate or extreme problems related to Pain/Discomfort and that half reported problems related to anxiety/depression. Our hypothesis, that longer length of stay in ICU and comorbidities (here as the presence of diabetes mellitus) were predictors of impaired HRQoL 1 year after ICU admission, was confirmed. However, our hypothesis that older age, male sex, and other comorbidities were predictors of impaired HRQoL was rejected.

The mean EQ‐5D‐3L value index of 0.83 in the current study was lower than the mean value index of 0.91 reported in the general Swedish population [33]. However, this is unsurprising considering that the participants in this study were affected by a severe disease requiring ICU treatment. Our median EQ‐5D‐3L value index of 0.87 were also lower than that of a prior study that reported a median value index of 0.91 [22]; here the differences could be attributed to the inpatient rehabilitation received by the participants of that study.

The most reported problems in our study were related to pain/discomfort and anxiety/depression, which is consistent with the findings from three other studies 1 year following COVID‐19 and ICU care [21, 22, 23]. Furthermore, more than one‐third of the participants in the current study reported problems related to Mobility and Usual Activities, which were higher than reported in a previous study [22]; here all the participants received inpatient rehabilitation after ICU care, which may explain the difference. In Self‐Care, every tenth participant experienced problems 1 year after COVID‐19 following ICU care, again consistent with prior research where 13% [19] and 9% [23] of study participants at 6 and 12 months after severe COVID‐19, respectively, experienced problems. However, this is lower than the previously reported 21% in another study 1 year after COVID‐19 following ICU care [22]. Due to the considerable prevalence of impaired long‐term HRQoL after ICU admission for COVID‐19, we suggest that long‐term individualized rehabilitation plans are required to support these individuals’ recovery.

The mean EQ‐VAS score of 67.6, interpreted as fair health [33], from our study was unsurprisingly lower compared to the mean score of 79.5 in the Swedish general population [33]. When comparing with previous research, also on first wave COVID‐19 ICU survivors, our EQ‐5D‐3L VAS scores were similar (67.6 vs. 68) [21]. Our EQ‐VAS median score of 70, though, was in line with two prior studies of COVID‐19, which also reported 70 as median score for the survivors 1 year after discharge from ICU [22, 23]. However, our EQ‐VAS median score was lower than a prior Swedish study's EQ‐VAS median score of 75, where the participants had a shorter ICU stay, which could indicate they had less severe COVID‐19 [36]. Interestingly, according to both the EQ‐5D‐3L and the EQ‐VAS, the HRQoL for the participants in our study was the same (a median EQ‐5D‐3L value index of 0.87 and a median EQ‐VAS score of 70) as in a Swedish 5‐year follow up in stroke survivors, although they had a substantially higher mean age of approximately 76 years [37]. Longer ICU stay has previously been shown to be a factor associated with impaired HRQoL 6 months after COVID‐19 following ICU care [19], which is consistent with the findings from the current study. Also, in the present study, longer ICU stay was found to be a significant predictor of problems related to usual activities and pain/discomfort. As previously stated, longer ICU stay was a highly probable proxy for a more severe degree of disease [36] which may explain these participants’ self‐perceived impaired HRQoL. Prior research has shown that self‐perceived HRQoL 3 months after ICU discharge, assessed using the EQ‐5D‐3L, was comparable between COVID‐19 survivors and non‐COVID‐19 survivors who had other critical illness, despite the longer ICU stay observed in the COVID‐19 group [38]. In addition, diabetes mellitus was found to be a significant predictor of increased problems related to Self‐Care at 1 year following COVID‐19. Individuals with diabetes mellitus may have complications such as reduced sensation in the lower extremity, impaired vision, and cardiovascular diseases [39, 40, 41], which may hinder their ability to self‐care and consequently lead to impaired HRQoL. In the present study, advanced age and male sex were identified as protective factors associated with Pain/Discomfort. This finding may be, albeit speculatively, attributed to the possibility that older individuals may have greater experience of pain and may employ different coping strategies to manage it and to that there may be sex‐related differences stemming from sociocultural and psychological factors, health conditions and disease patterns, supportive social networks and roles, as well as biological factors (e.g., hormonal influences). It is also likely that the large proportion of men (74.2%) in our study, influenced the outcome. Albeit using different assessment tools, there are similarities with a previous study of first wave COVID‐19 ICU survivors, showing female sex as a risk factor of impaired HRQoL [24].

The strengths of the current study lie in its consecutive inclusion, the well‐defined population from a large geographical area of Sweden and the long follow‐up period. The findings could be generalized to individuals cared for at an ICU for COVID‐19 during the first wave of the pandemic in Sweden and adds to the existing literature by providing country‐specific data on the long‐term prevalence of impaired HRQoL. However, there are several limitations to consider. The sample size was relatively small, which resulted in a limitation of the number of the included potential factors associated with impaired HRQoL. The results can only be generalized to these predictors quantified using the EQ‐5D‐3L. Furthermore, the five dimensions in EQ‐5D‐3L do not cover the whole range of HRQoL. The sample size increases the likelihood that true effects in subgroup analyses may be missed, even if they exist. There is also a risk that positive results may be found by chance when no true effect is present. Due to the sudden onset of the pandemic, data regarding the participants’ pre‐admission HRQoL were not available. To avoid recall bias, we chose not to collect this data retrospectively. Consequently, we are unable to ascertain how the participants’ HRQoL have been affected by either COVID‐19, their stay in the ICU or both. However, it is also possible that HRQoL remained unchanged compared to the pre‐COVID period. Moreover, we acknowledge that the observational nature of our study limits the ability to draw causal inferences from the outcomes. Several events may have had an impact on the participant's HRQoL in the year following their ICU admission. The participants’ HRQoL may have been affected by the rate of rehabilitation after ICU care, and we do not have any valid data of any intervention after discharge from the ICU. The moderate response rate (58%) may have influenced the results. However, we did not find any significant differences in the baseline characteristics of age, sex, or length of stay at ICU between the participants and the non‐participants, which is why we believe our results are likely to be representative and unbiased for this population. However, healthier individuals may have been overrepresented in the study due to the higher likelihood of them participating. Conversely, it is also possible that individuals with more severe health issues were more motivated to participate, given the study's focus on health impairments. This potential response bias should be considered when interpreting the results, as it may influence the generalizability of our findings. Another potential limitation is that some important variables that could have been associated with impaired HRQoL were not included. Higher BMI, for example, has previously been shown to be a predictor of impaired HRQoL after COVID‐19 [42]. Due to a substantial proportion of missing data, we were unable to analyse the BMI data in the current study. The absence of complete BMI data is a key limitation of this study, particularly in assessing its role as a predictor of HRQoL outcomes. Due to the extent of missing data, we did not use imputation methods to avoid potential biases (confounding and selection). As a result, the exclusion of BMI may limit the study's conclusions and introduce bias. Further studies with complete BMI data are needed to better evaluate its impact. Unfortunately, we did not have available data on other variables that may also influence HRQoL outcomes, such as socioeconomic status and mental health history.

We found that the greatest proportion of problems reported were related to pain/discomfort and anxiety/depression. Despite the large proportion of reported problems related to anxiety/depression, we did not find any predictors of this dimension. Further research with other potential predictors should have been merited, but is not possible more than more broadly, in terms of, for example, COVID‐19 vaccination. Furthermore, further research with a larger sample size and longer follow‐up time is recommended to determine whether HRQoL remains impaired or improves over time for individuals with severe COVID‐19 following ICU treatment. Similarly, the impact of BMI, socioeconomic status, and post‐ICU rehabilitation programs on HRQoL recovery after COVID‐19, for example, are other factors that could be of interest for future research.

## 5 Conclusion

Impaired HRQoL is common 1 year after COVID‐19 following ICU care. In this follow up, more than two‐thirds reported problems related to pain/discomfort and half reported problems related to anxiety/depression. Diabetes mellitus, longer stay in ICU, younger age and female sex were factors associated with impaired HRQoL. The significant long‐term prevalence of impaired HRQoL among individuals 1 year following ICU care due to COVID‐19 is a public health concern. Our findings imply that individuals who recovered after ICU admission for COVID‐19, and particularly those with diabetes mellitus, should be followed up beyond 1 year to identify those in need of mental and/or physical health care and rehabilitation. To support and enhance the recovery and HRQoL of COVID‐19 ICU survivors, the establishment of follow‐up clinics with multidisciplinary teams and the development of individualized care and rehabilitation plans seems worthy of serious consideration.

## Author Contributions

All the authors made substantial contributions to the concept. C.M.S. and C.U.P. performed the data collection, including the clinical examinations. C.M.S. drafted the manuscript. C.UP., N.H., and A.D. revised the manuscript critically. N.H. and C.U.P. performed the data analysis. C.U.P., the principal investigator of the GOT‐RECOV‐19‐ICU‐study, was responsible for the management of the data and takes complete responsibility for the integrity of the data and the accuracy of the data analysis. All authors read and approved the final version of the manuscript.

## Ethics Statement

This study was approved by the Swedish Ethical Review Authority (ID numbers: 2020‐03264 and 2021‐03220).

## Consent

Written informed consent was obtained from all participants.

## Conflicts of Interest

The authors declare no conflicts of interest.

## Transparency Statement

The lead author Carina U. Persson affirms that this manuscript is an honest, accurate, and transparent account of the study being reported; that no important aspects of the study have been omitted; and that any discrepancies from the study as planned (and, if relevant, registered) have been explained.

## Data Availability

The dataset is available from the principal investigator Carina U Persson (carina.persson@gu.se) following a reasonable request. According to Swedish regulations, permission to use data can be obtained after an application to and approval by the Swedish Ethical Review Authority. The datasets used and/or analysed during the current study are available from the corresponding author on reasonable request.
